# Long-term Quality of Life in Survivors of Brain Metastases: A Roller Coaster of Perspective

**DOI:** 10.7759/cureus.2358

**Published:** 2018-03-22

**Authors:** Naveen Kumar Reddy, Franklin C Brown, Miklos C Fogarasi, James B Yu, Judith Hess, Veronica S Chiang

**Affiliations:** 1 Department of Medicine, Frank H. Netter Md School of Medicine; 2 Neurology, Yale School of Medicine; 3 Medicine, Frank H. Netter Md School of Medicine; 4 Radiation Oncology, Yale School of Medicine; 5 Department of Neurosurgery, Yale School of Medicine; 6 Neurosurgery, Yale School of Medicine

**Keywords:** gamma knife radiosurgery, radiation oncology, long-term toxicity, stereotactic radiosurgery, breast cancer survivors, brain metastasis, quality of life

## Abstract

Longevity in cancer patients with brain metastases is increasingly being observed. This raises discussions about how best to maintain a good quality of life for these patients. Recent data suggest that post-treatment quality of life (QoL) can be maintained using new treatment options, but little data exist regarding the QoL in long-term survivors. This study of 19 patients surviving greater than two years from the initial treatment of brain metastases suggests that long-term QoL can be better than at the start of treatment and perhaps even better than normal, especially between three and five years post-treatment. This improved QoL seems mostly attributable to improved functional and social well-being and is possible as long as emotional and physical well-being are maintained within the normal range.

## Introduction

Quality of life (QoL) is becoming increasingly important as patients survive longer after treatment for cancer. This is particularly so for cancer patients who develop brain metastases. Whole brain radiation therapy (WBRT) and chemotherapy were previously the standard of care treatments, and the combination of these treatments, along with the progression of the disease itself, often affected neurocognitive function and therefore QoL [1-2]. Due to these increasingly recognized complications, the trend in the past decade has been to move towards radiosurgery for brain metastasis treatment and immunotherapy, where possible, for systemic treatment [3]. This seems to have resulted in less acute treatment-related toxicities as supported by recently published QoL data by Skeie et al. showing that the use of radiosurgery in conjunction with anti-cancer treatments did not negatively affect the QoL in patients with brain metastases in the first 12 months after stereotactic radiosurgery (SRS) [4]. The longer-term QoL outcomes, however, remain unknown and are the topic of this retrospective case series.

## Materials and methods

The Yale Gamma Knife database was queried for long-term survivors - defined as survival of greater than two years following initial diagnosis of brain metastasis. Of the 79 patients identified, 19 were willing to participate in testing. All participants completed the Functional Assessment of Cancer Treatment for Brain Tumors (FACT-Br) test in a single session [5]. Overall QoL scores, along with scores from the four individual components: physical well-being (PWB), functional well-being (FWB), emotional well-being (EWB), and social well-being (SWB), were then compared with normal and zero and the 12-month data from the SRS study by Skeie et al. discussed in the Introduction. The Kolmogorov-Smirnov normality test was applied to our case series data and the results were noted to be normally distributed. Therefore, the statistical significance of our findings was determined using the two-tailed T-test for parametric data.

## Results

Median patient age was 65.5 years (range: 50 - 88). Time from brain metastasis diagnosis to testing was 25.6 to 120.6 months (median: 60 months). The number of brain metastases treated ranged from one to 10 (median: 4). The mean total lesion volume treated was 14.1 mm^3^ (range: 0.6 - 39). Melanoma was the primary diagnosis in eight patients, lung cancer in six, and one patient each had renal, ovarian, breast, testicular, and rectal cancer. Six of the 19 patients were treated with immunotherapy only for their systemic disease, five received a combination of chemotherapy and immunotherapy, and eight received chemotherapy only. Three patients received WBRT early in their disease course. All patients were treated using radiosurgery during their course. QoL was not found to be significantly associated with any single clinical factor, including the type of systemic therapy, the form of brain radiation, or volume of brain metastases treated.

**Figure 1:**
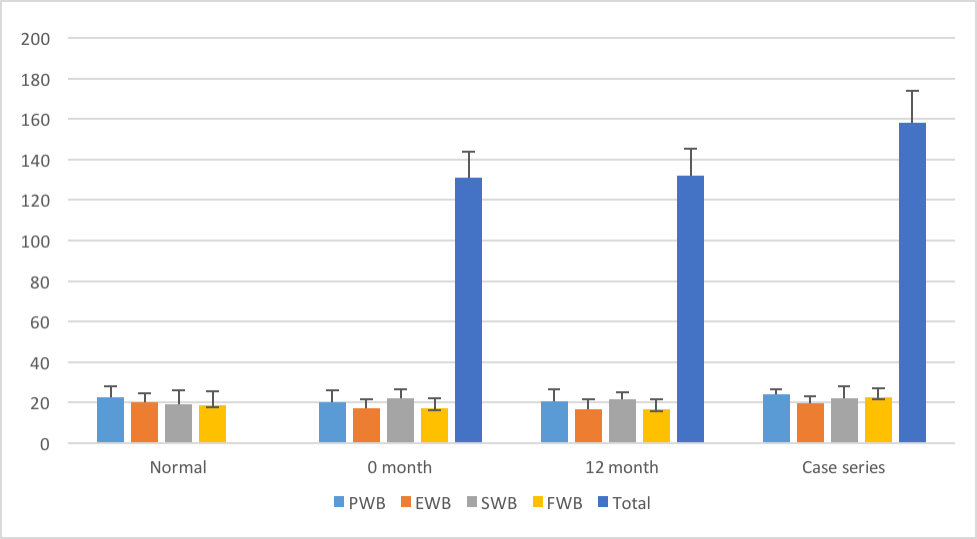
Total QoL and subcategory scores compared with normal and 0-12 month data QoL: quality of life; PWB: physical well-being; EWB: emotional well-being; FWB: functional well-being; SWB: social well-being

The mean overall total QoL score in the series cohort was 158.16 (standard deviation (SD) 24.8). This was significantly higher than the mean pre-treatment (zero - month) score of 130.9 (SD 22.7) and 12-month post-treatment score of 132.2 (SD 26.1), p = 0.0017 (Table 1). The mean PWB was 23.82 (case series cohort SD 2.74; normal mean: 22.7), EWB 19.82 (SD 3.26; normal mean: 19.9), SWB 21.88 (SD 5.99; normal mean: 19.1), and FWB 22.39 (SD 4.73; normal mean: 18.5) (Figure 1). The mean overall FWB scores were significantly higher in our cohort compared to the normal range (p = 0.0132), as well as when compared with zero and 12-month scores after SRS (p = 0.0004). The mean overall EWB scores were significantly higher in our cohort than zero and 12-month scores (p = 0.01), although not different than the normal range. The mean overall SWB and PWB scores were not different between our cohort, the normal range, and the zero and 12-month scores. 

**Table 1:** Raw QoL Scores QoL: quality of life; SD: standard deviation; PWB: physical well-being; EWB: emotional well-being; FWB: functional well-being; SWB: social well-being

	Normal	0 month cohort	12 month cohort	This case series	< 40 months	40 - 60 months	> 60 months
	Mean (SD)	Mean (SD)	Mean (SD)	Mean (SD)	Mean (SD)	Mean (SD)	Mean (SD)
PWB	22.7 (5.4)	20.2 (6.0)	20.5 (6.0)	23.82 (2.74)	23.00 (2.55)	25.50 (1.22)	22.83 (3.49)
EWB	19.9 (4.8)	17.2 (4.3)	16.4 (5.2)	19.82 (3.26)	19.20 (4.21)	19.83 (2.48)	20.33 (3.61)
SWB	19.1 (6.8)	22.3 (4.2)	21.6 (3.4)	21.88 (5.99)	24.20 (3.77)	24.17 (4.40)	17.66 (7.15)
FWB	18.5 (6.8)	17.1 (5.1)	16.5 (5.3)	22.39 (4.73)	20.80 (2.28)	26.50 (1.64)	19.61 (5.72)
Total	n/a	130.9 (22.7)	132.2 (26.1)	158.16 (24.81)	158.20 (12.99)	176.17 (20.73)	142.69 (25.89)

**Figure 2:**
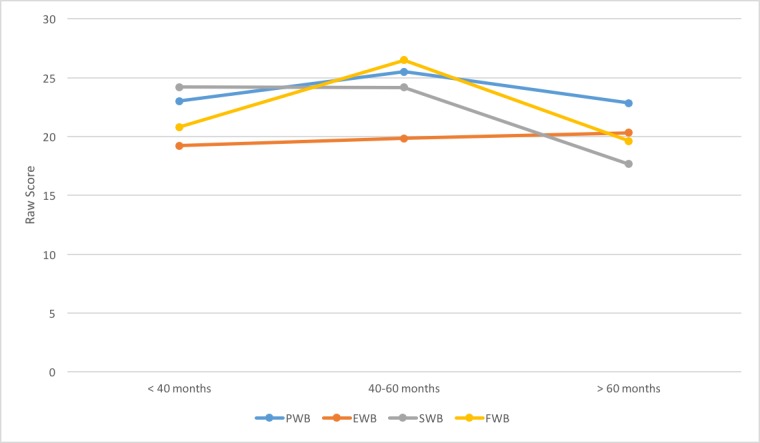
Changes in QoL subcategories over time QoL: quality of life; PWB: physical well-being; EWB: emotional well-being; FWB: functional well-being; SWB: social well-being

Hierarchical cluster analysis showed distinct clustering at three-time points within our data: (1) less than 40 months, (2) 40 - 60 months, and (3) greater than 60 months (Figure 2). Divided by clustered time points, the mean total QoL scores were 158.2 (SD 12.99) in patients tested at < 40 months (n = 6), 176.17 (SD 20.73) at 40 - 60 months (n = 7), and 142.69 (SD 25.89) at > 60 months (n = 6). The subcategory score that contributed most to the rise in QoL between 40 and 60 months was a mean FWB of 26.50 (SD 1.64) compared with a mean FWB of 20.80 (SD 2.88) at < 40 months, and 19.61 (SD 5.72) at the > 60-month time points. The return of QoL scores to normal at > 60 months was mostly attributable to a decrease in the mean SWB score to 17.66 (SD 7.15) compared with elevated mean SWB of 24.22 (SD 3.77) and 24.17 (SD 4.40) at < 40 and 40 - 60 months, respectively. The elevated SWB scores at <40 and 40 - 60 months trended towards being significantly higher than normal (p = 0.007). Minimal changes in PWB and EWB scores were seen across time (Figure 2). 

## Discussion

This case series represents a preliminary investigation into the QoL of long-term cancer survivors after treatment of brain metastases. While there are no normal ranges available for the total FACT-Br score, Skeie et al. recently published FACT-Br results for their study of 97 patients prior to and then for 12 months after radiosurgical treatment of brain metastases. Compared to their scores, the mean total QoL score in our cohort was 158.16 compared with their means of 130.9 and 132.2, although there was clearly an overlap between the ranges. Traditionally, a difference between means of greater than half a single standard deviation is considered likely significant; therefore, this data suggests that longer-term survival is associated with an improvement in the QoL.

When broken down into its components (PWB, SWB, EWB, and FWB) to try and determine what factors contribute most to total QoL, functional well-being was significantly higher than normal, while social well-being was trending upwards towards significance. SWB and FWB scores were particularly high in the 2-5 year post-treatment period, falling back into the normal range beyond five years. These results suggest that beyond two years after first treatment of their brain metastases, as long as their physical and emotional well-being is stable, long-term survivors feel better supported by their friends and family (SWB) and can work, sleep well, and enjoy their life (FWB). Also of note is that EWB was significantly better long-term post-treatment than in the first year of treatment as would be expected. This also contributed to the improved overall QoL scores in survivors. Beyond five years, all well-being scores returned to within normal range consistent with the possibility of being cured of cancer.

No specific clinical variables were found in this case series to be significantly associated with better or worse overall QoL. While WBRT and chemotherapy have been reported to affect QoL, this case series is limited by its small size. In addition, the interpretability of the results of this case series could be limited by several other concerns. The first is that QoL measures are usually obtained longitudinally, whereas in this case series, only single data time points are available. It is unknown if single time point data can be compared with longitudinal cohort data; future studies need to consider obtaining baseline scores prior to brain metastasis treatment, although the timing of the collection of this data relative to systemic disease course also needs to be considered. Secondly, it is unclear if it is reasonable to compare QoL results obtained in two different countries. Lastly, our cohort was obtained retrospectively and, therefore, those who agreed to undergo testing may represent a biased patient population with better QoL. Therefore, prospective QoL data collection from a much larger cohort is required to validate our findings.

## Conclusions

This case series suggests that in patients with brain metastases who have been successfully treated for their cancer and survived greater than two years, QoL appears to be higher than in the first year after brain metastasis diagnosis/treatment and was highest between three and five years after treatment. Based on subcategory analysis, good QoL scores were predominantly driven by high functional and social well-being between two and five years in patients who had normal physical and emotional well-being. This case series, therefore, suggests that the use of treatments that least affect physical and emotional well-being may then enable long-term brain metastasis survivors to achieve a quality of life that might be even better than normal. Larger cohort longitudinal studies are needed to confirm these findings.
